# Supplementary material for: Cloud BioLinux: pre-configured and on-demand bioinformatics computing for the genomics community
Source: BMC Bioinformatics. 2012 Mar 19;13:42. doi: 10.1186/1471-2105-13-42 (PMC3372431; doi:10.1186/1471-2105-13-42)
Supplement: Additional file 1 — Supplementary 1 Cloud BioLinux software documentation in the form of a mini, self-contained website. Users need to download and uncompress the .zip file, and open through a web browser the "index.html" file available on the main directory. (ZIP 1823 kb). [file 1471-2105-13-42-S1.ZIP › Cloud-BioLinux-Package-Documentation/docs/bl2seq.html]

Bio-Linux Software Documentation Pages

Back to search form

## bl2seq

|  |  |
| --- | --- |
| Name | bl2seq |
| Description | **bl2seq** (BLAST 2 Sequences) allows the blast searching of one sequence against another sequence, without having to run formatdb to create a blast database of the sequence to be searched against.  **bl2seq** is one of the programs distributed along with the old blastall program by the NCBI. The NCBI recommends that people start using the programs of the **blast+** package instead. Having said that, most documentation currently available still refers to blastall and the programs distributed with it.  The programs distributed with blastall include:  - bl2seq - blast2 - blastall - blastcl3 - blastclust - blastpgp - copymat - fastacmd - formatdb - formatrpsdb - impala - makemat - megablast - rpsblast - seedtop - taxblast  The man pages for these provide further details. ~ |
| Homepage | http://www.ncbi.nlm.nih.gov/BLAST/ |
| Remote Documentation | http://www.ncbi.nlm.nih.gov/Education/BLASTinfo/information3.html |
